# Lysophosphatidic acid counteracts glucagon-induced hepatocyte glucose production via STAT3

**DOI:** 10.1038/s41598-017-00210-y

**Published:** 2017-03-09

**Authors:** Evan P. Taddeo, Stefan R. Hargett, Sujoy Lahiri, Marin E. Nelson, Jason A. Liao, Chien Li, Jill K. Slack-Davis, Jose L. Tomsig, Kevin R. Lynch, Zhen Yan, Thurl E. Harris, Kyle L. Hoehn

**Affiliations:** 1 Department of Pharmacology, School of Medicine, University of Virginia, Charlottesville, VA 22908 USA; 2 Department of Microbiology, Immunology and Cancer Biology, School of Medicine, University of Virginia, Charlottesville, VA 22908 USA; 3 Department of Toxicology, School of Medicine, University of Virginia, Charlottesville, VA 22908 USA; 4 Robert M. Berne Cardiovascular Research Center, School of Medicine, University of Virginia, Charlottesville, VA 22908 USA; 5 School of Biotechnology and Biomolecular Sciences, University of New South Wales, Kensington, Sydney, NSW 2052 Australia

## Abstract

Hepatic glucose production (HGP) is required to maintain normoglycemia during fasting. Glucagon is the primary hormone responsible for increasing HGP; however, there are many additional hormone and metabolic factors that influence glucagon sensitivity. In this study we report that the bioactive lipid lysophosphatidic acid (LPA) regulates hepatocyte glucose production by antagonizing glucagon-induced expression of the gluconeogenic enzyme phosphoenolpyruvate carboxykinase (PEPCK). Treatment of primary hepatocytes with exogenous LPA blunted glucagon-induced PEPCK expression and glucose production. Similarly, knockout mice lacking the LPA-degrading enzyme phospholipid phosphate phosphatase type 1 (PLPP1) had a 2-fold increase in endogenous LPA levels, reduced PEPCK levels during fasting, and decreased hepatic gluconeogenesis in response to a pyruvate challenge. Mechanistically, LPA antagonized glucagon-mediated inhibition of STAT3, a transcriptional repressor of PEPCK. Importantly, LPA did not blunt glucagon-stimulated glucose production or PEPCK expression in hepatocytes lacking STAT3. These data identify a novel role for PLPP1 activity and hepatocyte LPA levels in glucagon sensitivity via a mechanism involving STAT3.

## Introduction

Blood glucose concentrations are maintained within a narrow range despite daily bouts of feeding and fasting. During fasting, hepatic glucose production (HGP) is vital for the maintenance of normal blood glucose levels^1^. However, with food intake HGP must be rapidly repressed to prevent hyperglycemia. Insufficient repression of HGP contributes to the pathophysiology of metabolic disorders including diabetes. Thus, defining the molecular mechanisms regulating HGP has important implications for understanding both normal physiology and potentially the development of new therapies for metabolic disorders associated with hyperglycemia.

HGP is regulated by numerous factors^2^, but the metabolic hormones glucagon and insulin have dominant roles. Glucagon is released from pancreatic alpha cells during fasting to increase HGP. Glucagon functions in part by stimulating expression of genes involved in gluconeogenesis, including *G6pc*, encoding glucose-6-phosphatase (G6Pase), and *Pck1*, encoding phosphoenolpyruvate carboxykinase (PEPCK)^3^. In contrast, insulin is secreted from pancreatic beta cells during feeding to repress *Pck1* and *G6pc* expression and HGP^3, 4^. In metabolic diseases such as type 2 diabetes, HGP is chronically elevated due to excess glucagon action and reduced insulin sensitivity, but the mechanisms underlying this imbalance are not fully defined. In particular, the bioactive lipid lysophosphatidic acid (LPA) is one molecule whose role in HGP remains controversial and unclear.

LPA levels are higher in diabetic patients than non-diabetic control subjects^5^, and higher in insulin resistant mice fed a high fat diet (HFD) compared to a normal diet^6, 7^. There is evidence that bolus injections of LPA can affect whole body glucose metabolism; however, these data are inconsistent and somewhat contradictory^6, 8^. For example, an acute injection of LPA at 50 mg/kg impairs insulin secretion and thereby reduces glucose clearance from the blood stream^6^, while another study reported that acute LPA injections between 3.3 and 6.6 mg/kg lowered blood glucose levels in the streptozotocin mouse model of type I diabetes that lacks insulin secretion^8^. Taken together, these data suggest that LPA may be beneficial for glucose homeostasis in the absence of insulin or during states of decreased insulin sensitivity. However, administration of the LPA receptor 1/3 inhibitor Ki16425 improved glucose tolerance in diet-induced obese mice concomitant with increased liver glycogen storage and reduced fasting levels of hepatic *Pck1* and *G6pc* ^6^. Based on these studies, it is difficult to reconcile the role of LPA in liver glucose metabolism. Furthermore, the role of endogenous LPA on HGP is entirely unknown.

LPA is an intermediate in the synthesis of glycerophospholipids, but is also a bioactive signaling molecule that binds to 6 known plasma membrane G protein coupled receptors (LPA receptors 1–6)^9, 10^ and at least one intracellular receptor^11^. LPA is primarily produced by decholination of lysophosphatidylcholine by the enzyme autotaxin, or by hydrolysis of an acyl chain of phosphatidic acid by phospholipase A enzymes^12^. LPA degradation is catalyzed by the integral membrane enzymes phospholipid phosphate phosphatases (PLPP), formerly known as lipid phosphate phosphohydrolases and phosphatidic acid phosphatases^13, 14^. PLPP1 and PLPP3 are ubiquitously expressed and found in the liver, while PLPP2 is not expressed in liver. Genetic deletion of PLPP3 in mice is embryonic lethal^15^, while knockout of PLPP1 results in healthy mice with no abnormal physiologic phenotype^16^. In this study, we investigated the roles of both exogenous and endogenous LPA in HGP by measuring glucose production in isolated primary hepatocytes treated with LPA and PLPP1 deficient hepatocytes and by assessing hepatic gluconeogenesis in PLPP1 KO mice.

## Results

### LPA attenuates glucagon-induced *Pck1 *expression and glucose production in wildtype primary hepatocytes

**Figure 1:**
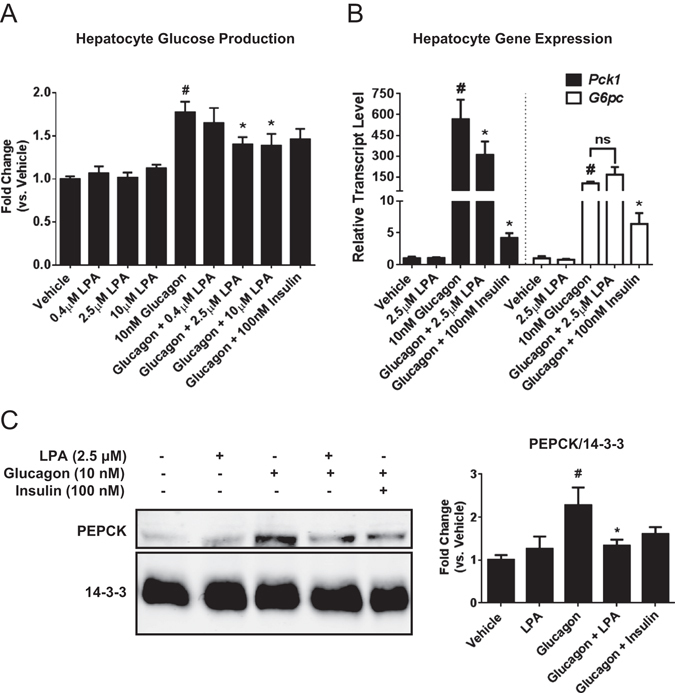
Exogenous LPA inhibits glucagon-stimulated glucose production in primary hepatocytes. (**A**) Basal and glucagon-stimulated glucose production from WT primary hepatocytes after 13 hrs in glucose-free DMEM in the absence or presence of LPA at 0.4, 2.5, and 10 μM; n = 5–6. Glucose released into the media (ng glucose/mg hepatocyte protein) was normalized to the vehicle control. (**B**) Gluconeogenic gene expression of WT hepatocytes after 13 hrs in glucose-free DMEM in the absence or presence of LPA (2.5 μM), glucagon (10 nM) and/or insulin (100 nM); n = 3. (**C**) Representative Western blot and quantification of PEPCK expression in WT primary hepatocytes treated for 13 hrs with LPA, glucagon and/or insulin, with 14-3-3 as a loading control; n = 4. All data are means ± standard error of the mean (SEM). For **A–C**, p < 0.05 by one-way ANOVA compared to vehicle control (#) or glucagon control (*). For **C**, the ANOVA was run with the following multiple comparisons: vehicle vs. glucagon and glucagon vs. glucagon + LPA. ns, not significant.

LPA is present in serum at concentrations between 300 nM and 10 μM^12, 17^. Specifically, 18:1 LPA is increased in high fat diet-fed mice^6^, suggesting that this particular LPA species may be important for regulation of hepatocyte glucose efflux. To investigate the role of exogenous LPA on hepatocyte glucose production, primary hepatocytes were isolated from C57BL/6 mice and treated with concentrations of 18:1 LPA spanning this physiologic range in the presence or absence of glucagon (Fig. 1A). LPA had no effect on basal hepatocyte glucose production at any concentration; however, LPA at 2.5 and 10 μM significantly decreased glucagon-induced glucose production (Fig. 1A). To determine how LPA reduced glucagon-stimulated glucose efflux, we investigated the effects of LPA on glucagon-induced gene expression of the rate-limiting enzymes in gluconeogenesis, *Pck1* and *G6pc*. LPA treatment alone had no effects on basal expression of *Pck1* or *G6pc*, but it selectively antagonized glucagon-mediated *Pck1* gene expression without altering *G6pc* (Fig. 1B). At the protein level, LPA completely prevented glucagon from increasing PEPCK protein expression (Fig. 1C). Notably, LPA was equally effective as insulin at blocking glucagon-mediated PEPCK protein expression (Fig. 1C).

### Deletion of PLPP1 increases endogenous LPA levels and inhibits hepatocyte *Pck1* expression and glucose production

**Figure 2:**
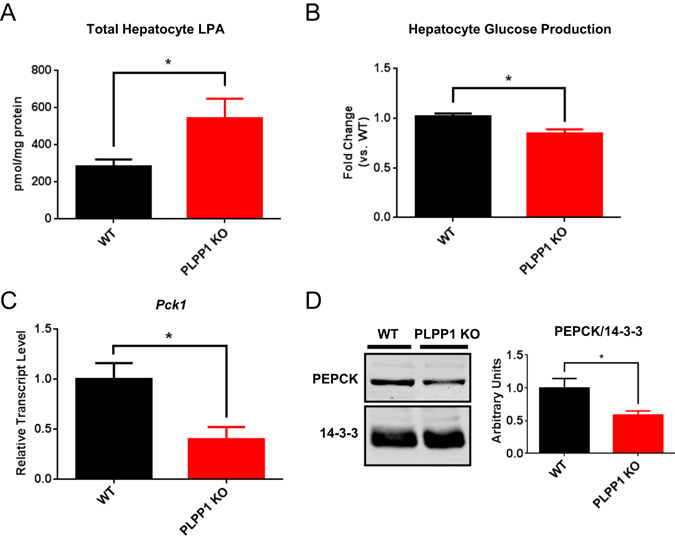
PLPP1 deletion in hepatocytes increases endogenous LPA concentrations and reduces hepatocyte glucose production. (**A**) Lipidomics analyses of total cellular lysophosphatidic acid (LPA) from PLPP1 KO and WT primary hepatocytes after overnight incubation in the presence of serum; n = 3. LPA levels are expressed as pmol/mg hepatocyte protein. (**B**) Basal glucose production (ng glucose/mg hepatocyte protein) from PLPP1 KO and WT primary hepatocytes, normalized to WT values; n = 20–21. (**C**) *Pck1* mRNA expression in isolated hepatocytes incubated overnight in the presence of serum; n = 3. (**D**) Representative blot and quantification of PEPCK protein expression in PLPP1 KO and WT primary hepatocytes incubated in serum-containing medium, with 14-3-3 as a loading control; n = 4. All data are means ± SEM. For A, *p < 0.05 by two-way ANOVA analysis of total and individual LPA species (shown in Supplementary Figure 1). For **B**–**D**, *p < 0.05 by two-tailed, unpaired t-test.

Next we determined the effects of endogenous LPA on hepatocyte glucose production in a cell-autonomous system. Primary hepatocytes were isolated from PLPP1-deficient mice and subjected to lipid extraction and LC/MS/MS to determine how PLPP1 deletion affected the abundance of LPA. These data showed that hepatocytes lacking PLPP1 had a 2-fold increase in total LPA levels (Fig. 2A). Lipidomic analysis of PLPP1 KO hepatocytes revealed increases in several species of LPA including 16:0, 18:0 and 18:1 LPA (Supplementary Figure 1). Concomitant with this elevation in LPA, PLPP1-deficient hepatocytes had a 15% reduction in glucose production compared to control hepatocytes (Fig. 2B). The reduced glucose release from PLPP1-deficient hepatocytes was associated with a 60% decrease in *Pck1* mRNA levels (Fig. 2C) and a 40% reduction in PEPCK protein (Fig. 2D) compared to control hepatocytes.

**Figure 3:**
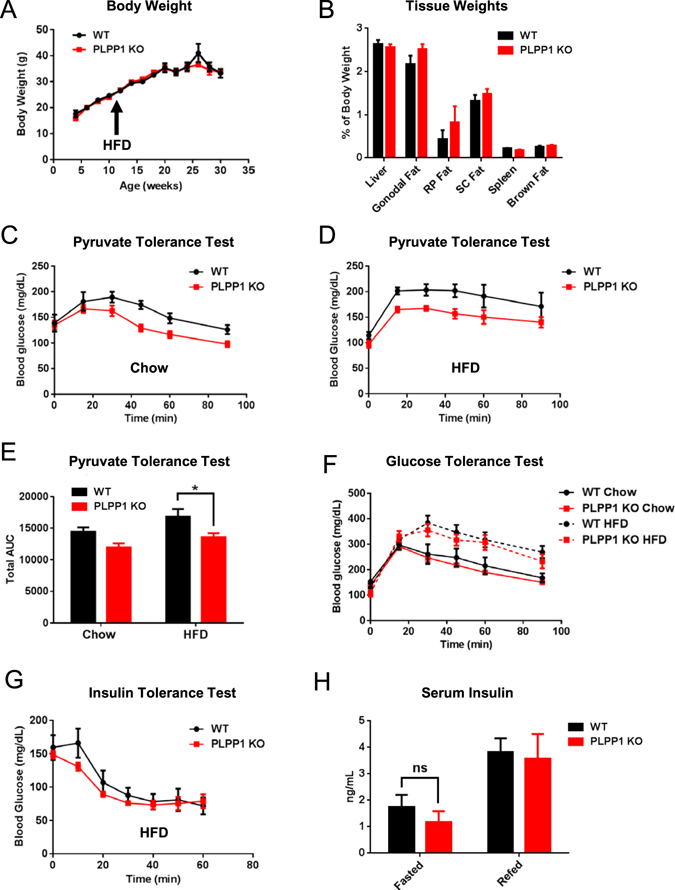
PLPP1 KO mice have reduced pyruvate-stimulated hepatic gluconeogenesis without changes in insulin or glucose tolerance. (**A**) Body weights of mice on chow diet and throughout the course of HFD feeding. Mice were fed a normal chow diet until approximately 12 weeks of age and were switched to a HFD for 18 weeks. Body weights were monitored weekly and averaged, yielding weight values for each mouse for every 2 weeks; n = 16–21 per genotype. (**B**) Weights of tissues harvested from HFD-fed mice, expressed as percentage of total body weight for each mouse. RP (retroperitoneal) and SC (subcutaneous) fat; n = 4–20 per genotype. (**C**,**D**) Pyruvate tolerance tests (PTTs) in overnight-fasted mice fed a chow or HFD diet for 15–17 weeks; for chow n = 6–10 per genotype and for HFD n = 7–8 per genotype. (**E**) Calculation of total area under the curve (AUC) from PTTs shown in (**C**,**D**). (**F**) Glucose tolerance tests (GTTs) administered to mice on chow diet or HFD for 15 weeks; for chow n = 8–11 per genotype and for HFD n = 7–8 per genotype. (**G**) Insulin tolerance tests (ITTs) administered to mice on HFD for 16 weeks; n = 4–5 per genotype. (**H**) Circulating insulin in the serum of overnight fasted and 2 hr refed mice on HFD; n = 6–7 per genotype. All data are means ± SEM. PTT (**C**,**D**), GTT (**F**) and ITT (**G**) were analyzed by repeated measures two-way ANOVA. For PTT AUC in **E**, *p < 0.05 by two-way ANOVA. For **H**, serum insulin was analyzed by two-way ANOVA. ns, not significant.

We next investigated the role of endogenous LPA on hepatic gluconeogenesis in PLPP1 KO mice. PLPP1 gene deletion did not alter body weight or body composition with either chow diet or high fat diet (Fig. 3A,B). However, PLPP1 KO mice fed high fat diet showed lower hepatic gluconeogenesis after bolus injection with pyruvate (Fig. 3C,D), as evidenced by significantly lower area under the PTT curve (Fig. 3E). In contrast, there were no genotype-specific differences in glucose tolerance (Fig. 3F), insulin tolerance (Fig. 3G) or circulating insulin levels (Fig. 3H), indicating that peripheral glucose clearance and insulin sensitivity in PLPP1 KO mice were comparable to control mice on high fat diet. Importantly, these data indicate that the decrease in glucose levels after pyruvate challenge in PLPP1 KO mice was not due to increased clearance of glucose. Furthermore, the effects on hepatic gluconeogenesis were not secondary to changes in liver glycogen, triglycerides, or cholesterol content as these parameters were unchanged between genotypes (Supplementary Figure 2A–C).

### Ablation of PLPP1 decreases hepatic *Pck1* expression *in vivo*

**Figure 4:**
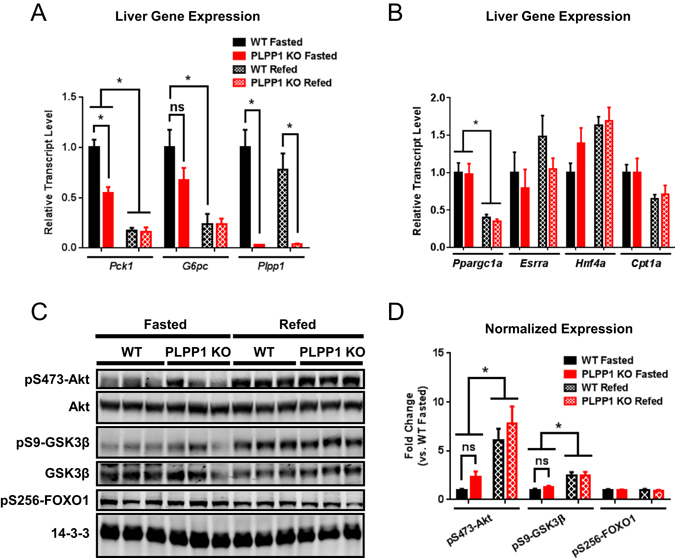
PLPP1 ablation reduces *Pck1* expression without altering major *Pck1* transcriptional stimulators or hepatic insulin signaling. (**A**) mRNA expression of *Pck1* (PEPCK), *G6pc* (G6pase) and *Plpp1* (encoding PLPP1) in liver tissue of HFD-fed WT and PLPP1 KO mice; n = 4–8 per genotype. (**B**) mRNA levels of *Ppargc1a* and *Hnf4a,* and their target genes *Esrra* and *Cpt1a* in livers of mice fed HFD; n = 4–5 per genotype. (**C**) Representative Western blots of insulin signaling pathway regulating gluconeogenic gene expression in the livers of mice fed a HFD, with 14-3-3 as a loading control. (**D**) Quantification of phospho-protein expression shown in (**C**), normalized to either total protein expression (pS473-Akt/Akt and pS9-GSK3β/GSK3β) or to 14-3-3 loading control (pS256-FOXO1); n = 4–5 per genotype. All data are means ± SEM. *p < 0.05 by two-way ANOVA. ns, not significant.

Consistent with the effects of LPA in primary hepatocytes, mice lacking PLPP1 had significantly lower expression of hepatic *Pck1* and a non-significant trend for lower *G6pc* expression in the fasted state (Fig. 4A). PLPP1 expression was not significantly regulated by fasting in wild type mice, and gene deletion was confirmed in liver tissue from KO mice (Fig. 4A). To elucidate how LPA decreased glucagon-stimulated PEPCK levels, we assessed the expression and activities of major transcription factors that drive *Pck1* transcription including *Ppargc1a* (PGC1α) and *Hnf4a* (HNF4α). However, neither the expression of these factors nor the expression of their downstream target genes including *Esrra* or *Cpt1a* was altered in a genotype-specific manner in the fed or fasted states (Fig. 4B). Because insulin signaling is a potent inhibitor of *Pck1* expression, we evaluated several key downstream signaling nodes including Akt, FOXO1, and GSK3β. However, no differences in insulin signaling were observed in the PLPP1 KO liver tissue in either the fasted or refed state (Fig. 4C,D).

### STAT3 is required for LPA inhibition of glucagon-stimulated *Pck1* expression and hepatocyte glucose production

**Figure 5:**
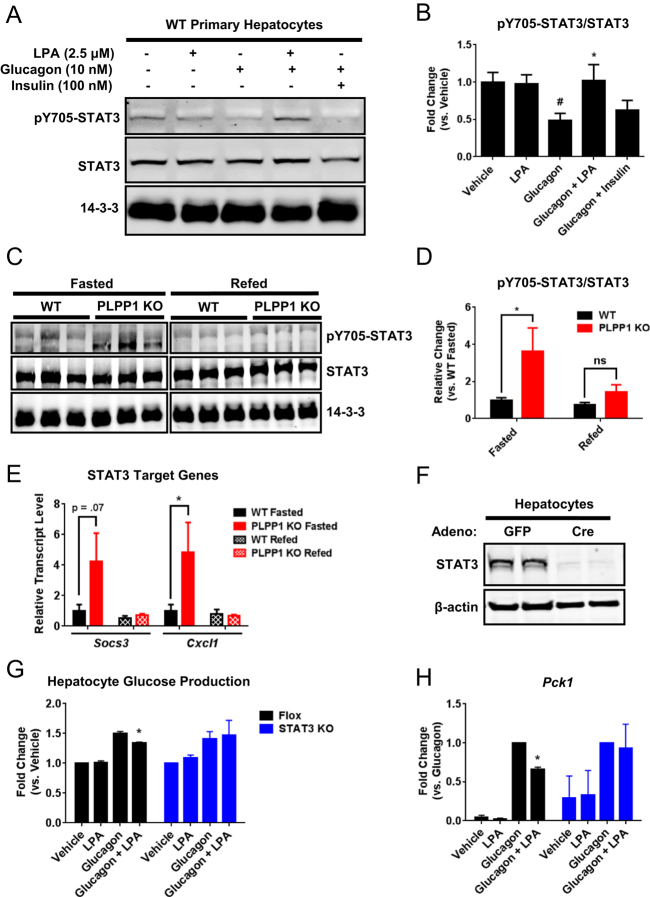
STAT3 is required for LPA inhibition of glucagon-stimulated hepatocyte glucose production. (**A**) Western blots of pY705-STAT3 and total STAT3 in WT primary hepatocytes incubated for 13hrs in glucose-free DMEM in the absence or presence of LPA (2.5 μM), glucagon (10 nM) and/or insulin (100 nM), with 14-3-3 as a loading control; representative blots from 5 independent experiments derived from 5 separate hepatocyte isolations. (**B**) Quantification of pY705-STAT3 normalized to total STAT3 expression; n = 5. (**C**) Representative Western blots of pY705-STAT3 and total STAT3 in livers of HFD-fed WT and PLPP1 KO mice in the fasted (overnight) or refed (2 hrs) state, with 14-3-3 as a loading control. (**D**) Quantification of pY705-STAT3 normalized to total STAT3 levels in mouse livers; n = 4–5 per genotype. (**E**) Hepatic expression of the STAT3 target genes *Socs3* and *Cxcl1* in HFD-fed mice; n = 4–5 per genotype. (**F**) Representative Western blots of STAT3 in isolated primary hepatocytes from STAT3 floxed mice 7 days after injection with either GFP control or Cre adenoviruses (Adeno). β-actin was used as a loading control. (**G**) Glucose production from Flox-GFP control or STAT3 KO primary hepatocytes incubated for 13hrs in glucose-free DMEM in the absence or presence of LPA (2.5 μM) and/or glucagon (10 nM); n = 3. (**H**) *Pck1* mRNA expression in Flox-GFP and STAT3 KO primary hepatocytes incubated in the absence or presence of LPA and/or glucagon for 13hrs; n = 3. All data are means ± SEM. For **B**, p < 0.05 by one-way ANOVA compared to vehicle control (#) or glucagon control (*). The ANOVA was run with the following multiple comparisons: vehicle vs. glucagon and glucagon vs. glucagon + LPA. For **D,E**, *p < 0.05 by two-way ANOVA. For **G,H**, *p < 0.05 by one-way ANOVA. ns, not significant.

Since LPA-mediated reduction in PEPCK expression was not associated with changes in major transcriptional drivers of PEPCK, we investigated the role of the transcription factor STAT3. STAT3 is a repressor of gluconeogenesis independently of insulin and PGC1α^18^. We found that treatment of WT primary hepatocytes with 2.5 μM LPA alone had no effect on STAT3 phosphorylation; however, LPA treatment prevented glucagon from decreasing STAT3 phosphorylation at tyrosine 705 (Fig. 5A,B), a critical residue for STAT3 transcriptional repression of PEPCK and gluconeogenesis^19^. Consistent with these data *in vitro*, liver tissue from fasted PLPP1 KO mice showed significantly higher phosphorylation of STAT3 at Y705 compared to WT mice (Fig. 5C,D). Increased STAT3 activity was observed in fasted liver tissue from PLPP1 KO mice compared to controls as evidenced by 4-fold and 4.8-fold increased expression of the STAT3 target genes *Socs3* and *Cxcl1*, respectively (Fig. 5E).

To determine if STAT3 was necessary for the inhibition of hepatocyte glucagon action by LPA, we generated STAT3-deficient hepatocytes by injecting STAT3 floxed mice with an adenovirus expressing Cre recombinase under the control of a liver-specific promoter for thyroxine binding globulin (TBG). An adenovirus expressing TBG-driven GFP was delivered to STAT3 floxed mice as a control. As expected, STAT3 expression was virtually absent in hepatocytes of mice administered the Cre virus (Fig. 5F). In STAT3 flox-GFP control hepatocytes, LPA decreased glucagon-induced glucose production (Fig. 5G) and both *Pck1* (Fig. 5H) and *G6pc* (Supplementary Figure 3) expression. However, LPA had no effect on glucose production (Fig. 5G) or gluconeogenic gene expression (Fig. 5H and Supplementary Figure 3) in hepatocytes lacking STAT3.

## Discussion

Hepatic glucose production (HGP) is a vital process that maintains a constant supply of glucose to the body during fasting. However, excess HGP can promote hyperglycemia and facilitate the pathogenesis of metabolic disorders including type 2 diabetes^20^. Therefore, it is important to fully understand the molecular regulation of HGP. LPA is a bioactive lipid that promotes cell proliferation, improves liver regeneration^21^, and may alter whole body glucose metabolism^6, 8^, but it is not known if endogenous LPA has a physiologic role in HGP. In this study, we show that ablation of the LPA-degrading enzyme PLPP1 increased hepatocyte LPA concentrations, decreased the expression of PEPCK, and reduced glucose production from primary hepatocytes. Similarly, fasted PLPP1 KO mice had reduced PEPCK expression and lower hepatic gluconeogenesis in response to a bolus of pyruvate. Notably, the decrease in pyruvate-stimulated gluconeogenesis was not secondary to changes in insulin sensitivity, glucose tolerance, or insulin levels as there were no genotype-specific differences in these parameters.

PEPCK expression is controlled at the gene level by multiple transcriptional co-regulators. Insulin is a potent repressor of *Pck1* expression, acting in part through phosphorylation and inhibition of the transcription factor FOXO1^2, 4^; however, we did not observe any evidence of increased insulin signaling in liver tissue from PLPP1 KO mice. We also investigated the potential role of well-known transcriptional activators of *Pck1* including *Ppargc1a* and *Hnf4a*, but we found no evidence that these factors contributed to the phenotype. In contrast, we did observe genotype-specific and LPA-specific effects on phosphorylation and activity of the *Pck1* transcriptional repressor STAT3. STAT3 activity was significantly higher in PLPP1-deficient liver tissues from fasted mice as evidenced by higher expression of STAT3 target genes *Socs3* and *Cxcl1*.

STAT3 is a well-known inhibitor of HGP. Activating phosphorylation of STAT3 drives its translocation to the nucleus where it binds to the promoters of *Pck1* and *G6pc* to inhibit their transcription^18, 22^. Cytokines including IL-6^23^ and IL-13^24^ suppress glucose production in a STAT3-dependent manner. Interestingly IL-6/STAT3 signaling is enhanced and glucose production reduced in mice heterozygous for the tyrosine phosphatase Ptpn2 which dephosphorylates STAT3^25^. Nie *et al.* discovered that nutrient status regulates STAT3 phosphorylation in a physiological manner, whereby fasting decreases pY705-STAT3 and feeding increases STAT3 phosphorylation in mice fed a normal chow diet^19^. The role of hepatic STAT3 was solidified by Inoue *et al.* ^18^ who showed that liver-specific ablation of STAT3 in mice results in higher expression of *Pck1*, while constitutively active STAT3 suppresses *Pck1* and HGP in mice. The STAT3 regulatory axis is disrupted in diabetes, as Kimura *et al.* have shown that endoplasmic reticulum stress blunts STAT3-mediated inhibition of glucose production in livers of db/db mice by reducing phosphorylation of STAT3^26^. Importantly, phosphorylation of STAT3 at Y705 is necessary for STAT3 to decrease both gluconeogenic gene expression and glucose production from hepatocytes^19^.

Interestingly, IL-6 can specifically direct STAT3 to inhibit *G6pc* transcription by blocking RNA polymerase II recruitment and decreasing histone H4 acetylation at the *G6pc* promoter^22^, a phenomenon not occurring on the *Pck1* promoter. Therefore, STAT3 could be directed to either the *G6pc* or *Pck1* promoter or both, depending on the upstream signaling stimuli. Indeed, this notion may help explain how LPA failed to decrease glucagon-stimulated *G6pc* expression in WT hepatocytes (Fig. 1B), but did significantly reduce glucagon-stimulated *G6pc* transcript levels in STAT3 Flox-GFP control hepatocytes (Supplementary Figure 3). The STAT3 Flox-GFP hepatocytes were exposed to adenoviruses which are known to activate an inflammatory response including IL-6^27^. It is possible that IL-6 or other cytokines produced as a consequence of adenovirus exposure may have restructured the transcriptional landscape on gluconeogenic gene promoters in such a way that sensitized *G6pc* to LPA/STAT3 signaling. The heterogeneity in LPA-mediated transcriptional regulation may also be due in part to the fact that STAT3 Flox-GFP and KO hepatocytes were isolated from older mice, and the physiological effects of LPA signaling may change with ageing^28^.

In WT hepatocytes, we observed that exogenous LPA counteracted the reduction in STAT3 phosphorylation mediated by glucagon. Importantly, we show that LPA required STAT3 to block glucose efflux and hepatic *Pck1* expression stimulated by glucagon. It is still unclear how LPA may stop glucagon from inactivating STAT3 during fasting to promote hepatocyte glucose production, but it is likely that LPA either impedes a glucagon-activated phosphatase that targets STAT3 or stimulates the phosphorylation of STAT3 which competes against glucagon-mediated dephosphorylation. However, at the concentrations used in this study, LPA treatment did not result in increased STAT3 phosphorylation. Thus, LPA most likely interrupts glucagon-mediated dephosphorylation of STAT3. Indeed, this may be the case, as glucagon activates the phosphatase calcineurin in the liver^29^, which can decrease Y705 phosphorylation of STAT3^30^.

LPA signaling engages several downstream mediators and therefore could intersect glucagon signaling via several mechanisms. LPA stimulates ERK1/2 phosphorylation^31, 32^, and may regulate glucose production via ERK activation, since the ERK1/2 phosphatase MKP3 stimulates gluconeogenic gene expression and glucose production^33^. However, the role of ERK1/2 in regulating HGP is still uncertain, given that ERK2 can also stimulate PEPCK expression^34^. LPA can also stimulate activating phosphorylation of Akt^35, 36^, a well-established mediator of insulin inhibition of gluconeogenesis at the transcriptional level. Yet, the LPA/Akt pathway seems to primarily affect cell survival, proliferation and migration^36^. In our study, we did not observe any changes in Akt phosphorylation in livers of fasted PLPP1 KO mice exhibiting reduced gluconeogenesis by PTT. We demonstrate that 18:1 LPA reduces hepatocyte glucose production and *Pck1* through STAT3. Of note, 18:1 LPA is increased in diet-induced insulin resistant mice^6^ and is one of the most abundant hepatic LPA species in mice^37^. Despite this, it is still possible that different molecular species of LPA could exert anti-gluconeogenic actions through multiple pathways since PLPP1 deletion in hepatocytes also increased 16:0 and 18:0 LPA. Future studies are required to identify the precise pathways whereby LPA intersects glucagon signaling and which of these may depend on STAT3.

It is intriguing that a mild reduction in glucose production was observed in PLPP1-deficient primary hepatocytes in the basal state, while exogenous LPA had no effect on basal glucose production in WT primary hepatocytes. Instead, exogenous LPA decreased glucagon-stimulated PEPCK expression and glucose release in WT primary hepatocytes. PLPP1 is localized at both the plasma membrane and internal membranes^15^, thus it is worthwhile to note that administration of exogenous LPA may also increase intracellular LPA because the monoacylglycerol produced by hydrolysis of LPA at the cell surface can cross the plasma membrane and be rephosphorylated to LPA inside cells^13^. Intracellular LPA can activate nuclear receptor LPA1^11, 38^ and directly activate PPARγ^39^, but the consequences of these intracellular actions of LPA on HGP have not been elucidated. Thus, one possible explanation is that LPA may antagonize glucagon action through extracellular and/or intracellular mechanisms. Another intriguing finding was that exogenous LPA had more potent effects at preventing glucagon-induced PEPCK protein expression than *Pck1* gene expression. It is possible that LPA could regulate steady state PEPCK protein levels by disrupting the balance between degradation-promoting acetylation by p300 and stabilizing deacetylation by Sirt2^40^. However, additional experiments are required to determine whether LPA also affects PEPCK stability.

In summary, this study identifies a new and important role for endogenous LPA in hepatocyte glucose production. We identified that PLPP1 deletion increased physiologic concentrations of LPA in hepatocytes concomitant with reduced glucose production. PLPP1 deletion was not sufficient to protect from diet-induced insulin resistance compared to control mice. Although one limitation of this study is that we did not have age-matched 30-week old chow-fed control mice, PLPP1 KO and control mice fed HFD had nearly identical glucose and insulin tolerance. Even though PLPP1 deletion had a mild effect on metabolic phenotypes, it remains possible that inhibition of other PLPP family members may have additional effects on increasing LPA concentrations and driving more potent repressive effects on glucose production. One possibility is that LPA may increase in diabetes as a compensatory mechanism to reduce uncontrolled gluconeogenesis in the face of insulin resistance. The mechanism linking PLPP1 and LPA to decreased hepatocyte glucose production is most closely tied to reduced transcriptional expression of *Pck1*and reduced PEPCK protein; however, one limitation of this study is that PEPCK enzyme activity was not determined. Finally, there are a few caveats to consider if this LPA-based mechanism is to be harnessed as a therapeutic strategy. While genetic deletion of PLPP1 in mice is not associated with any adverse phenotype detected by us and others^16^, targeting PLPP1 to increase LPA concentration will need to be carefully considered in the context of unwanted consequences. Paradoxically, LPA is known to potentiate cAMP/PKA signaling^12, 36^ and increase intracellular calcium via IP_3_^11^; two factors that promote glucose production. Furthermore, very high acute concentrations of LPA can inhibit insulin secretion^6^, and excess LPA is shown to facilitate increased cell proliferation and tumorigenesis in certain cancers^41, 42^. In the scenario that inhibition of PLPP1 is too risky for an anti-diabetes therapy, the information learned from this study can be used to investigate other potential targets in this pathway downstream of LPA.

## Materials and Methods

### Materials

Glucagon, dexamethasone, bovine serum albumin (BSA), and lysophosphatidic acid (LPA, 18:1) were purchased from Sigma Aldrich (St. Louis, MO). Free fatty acid-free BSA was purchased from ThermoFisher (Waltham, MA). Amplex Red was purchased from Invitrogen (Carlsbad, CA). Insulin was obtained from EMD Millipore (Billerica, MA) and Novo Nordisk (Bagsvaerd, Denmark). Primary antibodies for phospho-Akt (S473), total Akt, phospho-GSK3β (S9), total GSK3β and phospho-FOXO1 (S256) were obtained from Cell Signaling (Beverly, MA). Antibodies against phospho-STAT3 (Y705) and total STAT3 were purchased from BD Biosciences (San Jose, CA). The antibodies for PEPCK and 14-3-3 were purchased from Santa Cruz Biotechnology (Dallas, TX). The β-actin antibody was obtained from Sigma Aldrich. Goat anti-mouse IgG (DyLight 800 conjugate) and goat anti-rabbit IgG (DyLight 680 conjugate) polyclonal secondary antibodies were obtained from Rockland (Limerick, PA). GFP (AAV8.TBG.PI.eGFP.WPRE.bGH) and Cre (AAV8.TBG.PI.Cre.rBG.) viruses were purchased from the Penn Vector Core, Perelman School of Medicine, University of Pennsylvania (Philadelphia, Pennsylvania).

### Isolation and culture of primary hepatocytes

Primary mouse hepatocytes from PLPP1 knockout (KO), WT control, STAT3 liver knockout (LKO) and STAT3 floxed mice were isolated by collagenase perfusion as previously described^43^. The perfused liver was excised and immediately placed in ice-cold sterile 1x PBS. The liver was rinsed and placed into cold plating medium (DMEM supplemented with 25 mM glucose, 10% FBS, 4 mM L-glutamine, 1 μM dexamethasone, 100 nM insulin and 1% pen/strep). The liver lobes were gently scored with sterile fine-point forceps, and the liver was repeatedly shaken in the plating medium to release hepatocytes into the medium. The hepatocyte suspension was filtered through a 70 μm cell strainer (Corning Life Sciences) into a 50 mL tube and centrifuged at 50 × g for 3 min at 4 °C. The supernatant was aspirated, and the cell pellet was resuspended in 10 mL of cold plating medium and mixed with 10 mL of 90% Percoll (Sigma) in sterile PBS. The resuspended cells in Percoll were centrifuged at 100 × g 6 min at 4 °C, and the supernatant was aspirated. The cells were washed by resuspending in 20 mL of cold plating medium and centrifuging at 50 × g for 3 min at 4 °C. The supernatant was aspirated and the pellet was fully resuspended in 25–35 mL of warm plating medium. Viable hepatocytes were counted by taking 50 μL of resuspended cells and mixing with 50 μL of Trypan blue. Viability was assessed by Trypan blue exclusion and was ≥90% for each isolation. Primary hepatocytes were seeded in collagen-coated 6- or 12-well plates or 6 cm dishes in plating medium at 1.5 × 10^5^ cells/mL. Four hours after seeding, hepatocytes were washed 1x with PBS, and incubated overnight in serum-free medium (DMEM with 5 mM glucose, 0.2% BSA and 1% pen/strep) prior to hepatocyte glucose production and hormone/LPA-stimulated gene and protein expression experiments. For RNA and protein isolation and LPA measurement in PLPP1 KO and WT control hepatocytes, cells were incubated overnight in serum-containing medium (DMEM with 5 mM glucose, 10% fetal bovine serum and 1% pen/strep) and given fresh serum-containing medium for 3 hrs prior to harvest.

### Hepatocyte glucose production assays

Hepatocyte glucose production assays were performed as previously described in primary mouse hepatocytes^44^ with minor modifications. Primary hepatocytes were seeded in 12-well plates at 1.5 × 10^5^ cells/well and cultured at 37 °C 5% CO_2_ overnight in serum- and hormone-free medium. After serum/hormone starvation, hepatocytes were washed twice with sterile PBS, and incubated in 400 μL/well of glucose production buffer (serum-, glucose-, and phenol red-free DMEM with 3.7 g/L sodium bicarbonate, 0.6% BSA, 10 mM HEPES, 2 mM sodium pyruvate, and 20 mM lactic acid, pH 7.4) and incubated at 37 °C for 13 hrs in the absence or presence of glucagon, insulin or LPA. For experiments measuring HGP in the presence of LPA, cells were incubated with glucose production buffer containing free fatty acid-free BSA. Cells were immediately placed on ice, and the medium from each well was harvested and centrifuged at 700 rpm for 5 min. Glucose released into the medium was detected using an Amplex Red/Glucose Oxidase kit (Invitrogen) and quantified spectrophotometrically at 560 nm with an Infinite M200 plate reader (Tecan). Glucose levels in the medium were normalized to total cellular protein content in each well.

### Real-time quantitative reverse transcription PCR (qRT-PCR)

Measurement of gene expression by qRT-PCR was performed as previously outlined^43^. Briefly, total RNA was isolated using Trizol or Direct-zol (Zymo Research) and used for cDNA synthesis (2 μg liver RNA or 1–2 μg cell RNA) by two-step RT-PCR with the High Capacity cDNA synthesis kit (Roche). Levels of mRNA were semi-quantified with Sensifast SYBR Green mix (Bioline) and gene-specific primers (Integrated DNA Technologies) and were normalized to the housekeeping gene *Ppia* (CypA). For specific primer sequences, see Supplementary Table 1.

### Western blotting

Following treatment, cells were washed twice with ice-cold PBS and lysed with HEPES–EDTA–Sucrose lysis buffer (250 mM sucrose, 20 mM HEPES pH 7.4, and 1 mM EDTA) containing 2% SDS or RIPA buffer [150 mM NaCl, 10 mM NP-40, 0.5% sodium deoxycholate, 0.1% SDS, 50 mM Tris pH 7.5] containing protease inhibitors (Roche) and phosphatase inhibitors (2 mM Na-orthovanadate, 1 mM Na-pyrophosphate, 10 mM Na-fluoride, 250 nM microcystin LR). Whole cell lysates were sonicated and cleared of insoluble material by centrifugation^45^. Frozen livers (15–30 mg) were homogenized in 20× volumes (~400–600 μL) of RIPA buffer supplemented with protease inhibitors (Roche) and phosphatase inhibitors. Livers were homogenized with a hand held homogenizer (Kimble-Chase). Homogenates were sonicated, rotated at 4 °C for 1 hr and centrifuged at 16,000 xg at 4 °C for 10 min. Lysates were diluted in 4× Laemmli buffer and denatured at 65 °C for 5 min. Cellular proteins (20–40 μg) were resolved on 8–10% SDS-polyacrylamide gels and electro-transferred for 80–105 min at 4 °C onto nitrocellulose membranes. Equal protein loading was confirmed by Ponceau staining. Protein expression was detected with the following primary antibodies: phospho-Akt S473 (1:1000), total Akt (1:1000), phospho-GSK3β S9 (1:1000), total GSK3β (1:1000), phospho-FOXO1 S256 (1:1000), PEPCK (1:500), phospho-STAT3 Y705 (1:500), total STAT3 (1:1000), 14-3-3 (1:1000) and β-actin (1:1000). Primary antibodies were detected using goat anti-mouse IgG (DyLight 800 conjugate) or goat anti-rabbit IgG (DyLight 680 conjugate) polyclonal secondary antibodies (1:8000). Membranes were visualized, and protein band intensities quantified, using the LI-COR ODYSSEY System and software (LI-COR, Lincoln, NE).

### Animals

Food and water were provided *ad libitum* until the date of study and all animal care was in compliance with NIH guidelines and approved by the University of Virginia Animal Care and Use Committee. The high fat diet (45% kcal as fat) was purchased from Research Diets (D12451). Normal chow diet was purchased from Harlan Teklad (diet 7912). Animals were maintained on a 12 hr/12 hr light/dark schedule at 68–72 °F and housed 4–5 per cage. Generation of PLPP1 hypomorph (PLPP1 KO) mice, which lack PLPP1 expression in all tissues except the brain, has been previously described^16, 41^. PLPP1 KO mice were maintained on a C57BL/6 background. Liver STAT3 KO (LKO) mice were generated by injecting 17 week old STAT3 floxed mice with GFP control or Cre adenoviruses (1 × 10^11^ genome copies/mouse) via tail vein administration, as previously described^46^. Hepatocytes were isolated from STAT3 LKO mice for experiments 7 days post-injection when hepatocyte STAT3 loss was confirmed by Western blotting. Glucose, insulin and pyruvate tolerance tests were performed as described^43, 47^ in mice fasted 5–6 hrs (glucose and insulin tolerance) or overnight (~16 hrs, pyruvate tolerance). Mice were fasted overnight (~16 hrs) or fasted and refed for 2 hrs with HFD prior to euthanasia and harvesting of tissues for biochemical analyses.

### Serum and tissue analyses

Serum insulin was determined by ELISA. Briefly, a 96 well plate was coated overnight at 4 °C with a mouse monoclonal anti-insulin antibody (E86210M, at 0.9 μg/mL) and blocked for 1 hr at room temperature. Insulin standards and mouse serum samples (10 μL) were added to the plate and incubated with a guinea pig anti-insulin antibody (Linco) for 1 hr at room temperature on an orbital shaker, then at 4 °C overnight. Wells were incubated with a biotin-conjugated donkey anti-guinea pig IgG secondary antibody (1:10,000) at room temperature for 1–2 hrs, followed by addition of streptavidin-horseradish peroxidase (1:10,000, Zymed) for 30–60 min at room temperature. Wells were incubated with Ultra-TMB for 30 min, before addition of stop solution (0.18 N H_2_SO_4_) and spectrophotometric measurement at 450 and 590 nm. Triglycerides were measured in liver tissue by colorimetric assay (Pointe Scientific, Canton, MI). Liver cholesterol was quantified by colorimetric assay (Infinity Cholesterol Liquid Reagent, Thermo Scientific), according to manufacturer’s instructions. Glycogen content in livers was assessed following previously published methods^47^.

### Measurement of hepatocyte LPAs

LPAs in mouse primary hepatocytes were measured via liquid chromatography mass spectrometry, as previously described^47^, with the following modifications. Primary hepatocytes in 6 cm dishes were harvested in 200 μL/dish of ice-cold PBS and sonicated. Protein concentrations were determined by BCA assay. Equal volumes of hepatocyte homogenates (75 μL/sample) were diluted and mixed in acidified methanol (0.1 N HCl) containing an internal standard cocktail for glycerolipids (50–100 nM C15-diacylglycerol and C17-lysophosphatidic acid). Extracted glycerolipids were resuspended in solvent containing 69% methanol and 31% millipure H_2_O (v/v) supplemented with 10 mM ammonium acetate. LPAs were analyzed after separation with a Supelco C18 column. Mobile phase A consisted of 60% acetonitrile, 40% H_2_O and 0.1% formic acid with 1 mM ammonium formate. Mobile phase B consisted of 90% isopropanol, 10% acetonitrile and 0.1% formic acid with 1 mM ammonium formate. Total flow rate was 0.6 mL/min for LPAs. Hepatocyte LPAs were normalized to C17-LPA internal standard and expressed as pmol LPA adjusted to protein concentrations for each sample.

### Statistics

Data were expressed as means ± standard error of the mean (SEM) of at least 3 independent experiments or animals per group. p-values were calculated by two-tailed unpaired t-test, one-way ANOVA with Dunnett’s, Sidak’s or Holm-Sidak’s post-hoc test, two-way ANOVA with either Tukey’s or Sidak’s post-hoc test and repeated measures two-way ANOVA with Sidak’s post-hoc test. Statistical significance was set at p < 0.05.

## Electronic supplementary material

Supplementary Information
