# Supplementary material for: Lysophosphatidic acid counteracts glucagon-induced hepatocyte glucose production via STAT3
Source: Sci Rep. 2017 Mar 9;7:127. doi: 10.1038/s41598-017-00210-y (PMC5428006; doi:10.1038/s41598-017-00210-y)
Supplement: Supplementary file 1 — Supplementary Information [file 41598_2017_210_MOESM1_ESM.pdf]

Supplementary Information

Lysophosphatidic acid counteracts glucagon-induced hepatocyte glucose production via STAT3

Evan P. Taddeo[1], Stefan R. Hargett[1], Sujoy Lahiri[1], Marin E. Nelson[1], Jason A. Liao[1], Chien Li[1], Jill K. Slack-Davis[2], Jose L. Tomsig[3], Kevin R. Lynch[1], Zhen Yan[1,4], Thurl E. Harris[1], and Kyle L. Hoehn[1,5*]

Departments of Pharmacology[1], Microbiology, Immunology and Cancer Biology[2], and Toxicology[3], and the Robert M. Berne Cardiovascular Research Center[4], School of Medicine, University of Virginia, Charlottesville, VA, 22908; and the School of Biotechnology and Biomolecular Sciences, University of New South Wales, Kensington, Sydney, NSW 2052, Australia[5]

*To whom correspondence should be addressed: Dr. Kyle L. Hoehn, School of Biotechnology and Biomolecular Sciences, University of New South Wales, Sydney, NSW 2052, Australia, Telephone: (+61 2) 9385 9399, FAX: (+61 2) 9385 1483, Email: k.hoehn@unsw.edu.au

Supplementary Table 1. Primer sequences used in real-time qRT-PCR analyses.

Gene	Forward (5’ to 3’)	Reverse (5’ to 3’)
Cpt1a	TTGGGCCGGTTGCTGAT	GTCTCAGGGCTAGAGAACTTGGAA
Cxcl1	CCGAAGTCATAGCCACACTCAA	GCAGTCTGTCTTCTTTCTCCGTTAC
Esrra	CCTCCCGCCTTCTACAGGT	CACACGGCACAGTAGCGAG
G6pc	CCGGATCTACCTTGCTGCTCACTT	TAGCAGGTAGAATCCAAGCGCGAAAC
Hnf4a	TGCAGGTTTAGCCGACAATG	TCCTTCTTCATGCCAGCCC
Pck1	CCACAGCTGCTGCAGAACAC	GAAGGGTCGCATGGCAAA
Plpp1	TCACGGAACTACTCAACCAATC	AGCAGGAAGTAATACGCATCC
Ppargc1a	CCCTGCCATTGTTAAGAC	TGCTGCTGTTCCTGTTTT
Ppia	CGATGACGAGCCCTTGG	TCTGCTGTCTTTGGAACTTTGTC
Socs3	GCTCCAAAAGCGAGTACCAGC	AGTAGAATCCGCTCTCCTGCAG

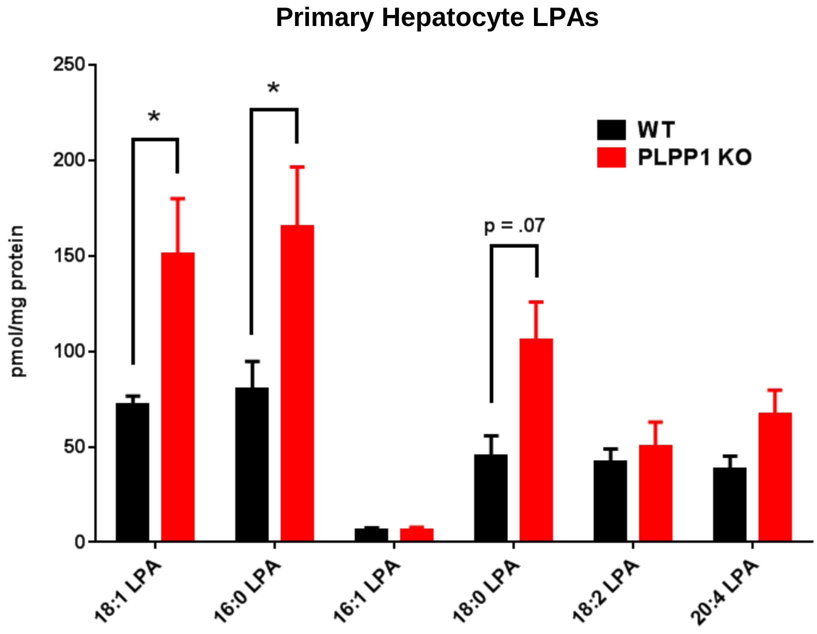

Supplementary Figure 1. Lipidomic profiling of LPA species in primary hepatocytes from PLPP1 KO and WT mice. Primary hepatocytes were isolated from PLPP1 KO and WT control mice and incubated overnight in serum-containing media. Hepatocytes were incubated for 3hrs in fresh serum-containing media, washed 2x in PBS and were harvested for lipidomic measurement of LPAs, as described in the Methods. LPAs are expressed as pmol/mg hepatocyte protein. n = 3 independent experiments. All data are means ± SEM. *p < .05 by two-way ANOVA.

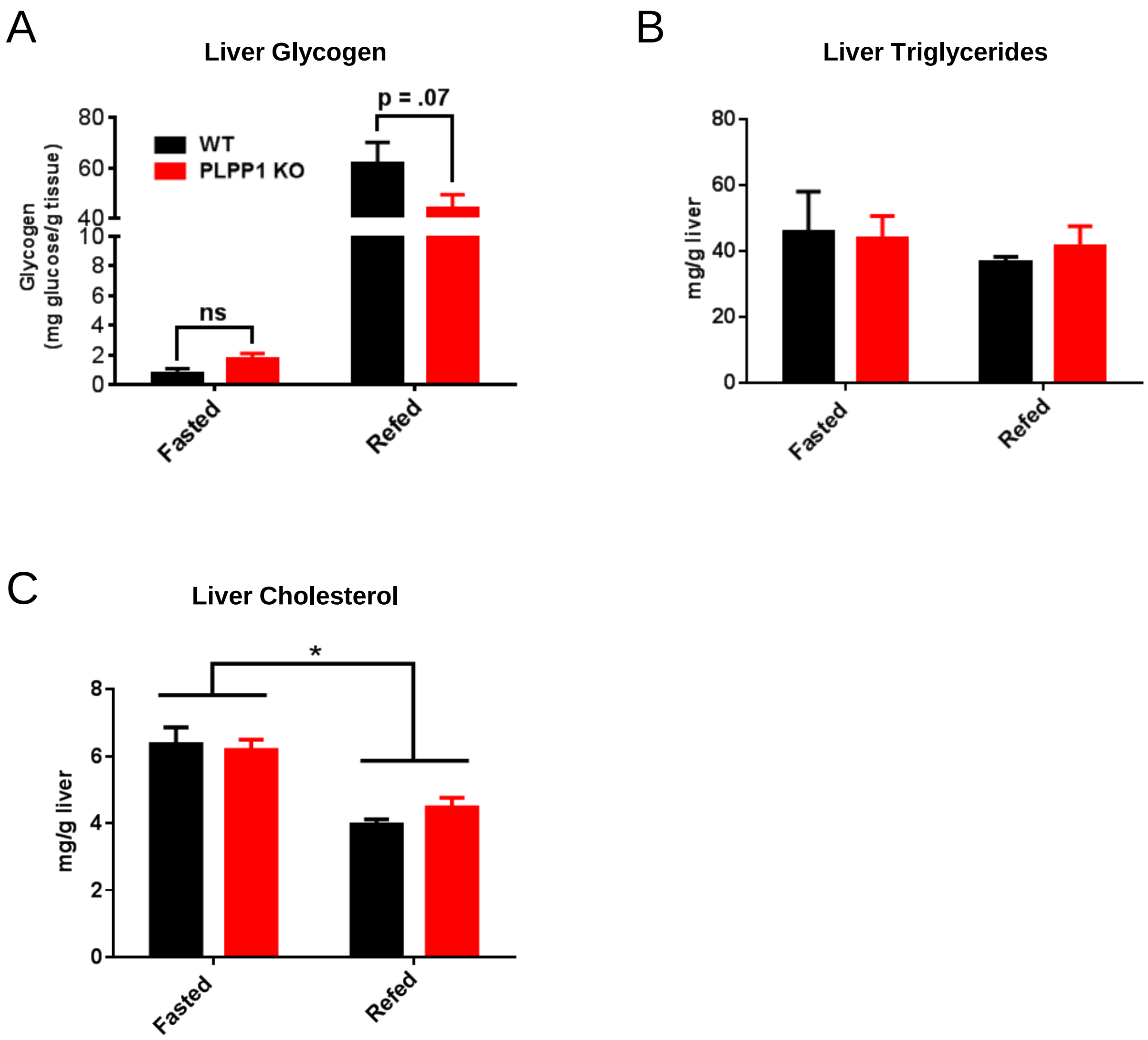

Supplementary Figure 2. Liver glycogen and lipid content of PLPP1 KO and WT mice on HFD. (A) Hepatic glycogen content in overnight-fasted or refed animals. Glycogen was extracted from liver tissue, digested into free glucose, and glycogen-derived glucose was measured spectrophotometrically. (B) Triglyceride content and (C) cholesterol levels in livers of HFD-fed mice. n = 4-5 per genotype. All data are means ± SEM. For A, liver glycogen was analyzed by two-way ANOVA. For C, *p < .05 by two-way ANOVA. ns, not significant.

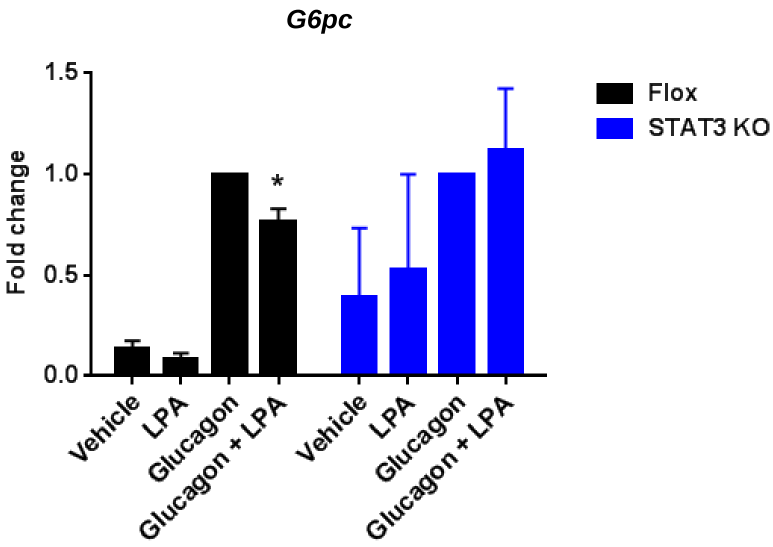

Supplementary Figure 3. Expression of *G6pc* mRNA in LPA-treated STAT3 KO and Flox control primary hepatocytes. STAT3 KO and Flox-GFP control primary hepatocytes were incubated in the absence or presence of 10nM glucagon and/or 2.5µM LPA for 13hrs. Cells were then harvested for RNA, and *G6pc* mRNA expression was assessed by qPCR; n = 3 independent experiments from 3 separate hepatocyte isolations. All data are means ± SEM. *p < .05 by one-way ANOVA.
